# Microbe: Are We Ready for the Next Plague?

**DOI:** 10.3201/eid1111.051084

**Published:** 2005-11

**Authors:** Peter B. Jahrling

**Affiliations:** * National Institutes of Health, Bethesda, Maryland, USA

**Keywords:** book review, SARS, West Nile Virus, plague

Microbe: Are We Ready for the Next Plague? by Alan Zelicoff and Michael Bellomo is a comprehensive, yet succinct, account of the threat to public health posed by microbial pathogens. What distinguishes this book from the surfeit of recent books hyping the threat of bioterrorism are its balanced perspective and elucidation of naturally emerging disease threats, such as severe acute respiratory syndrome (SARS) or West Nile virus, as exotic entities requiring a rapid and effective response; Mother Nature is quite the bioterrorist herself. Early recognition that an event has occurred is key to containment of the nascent epidemic.

The authors provide sufficient basic science background to bring the uninitiated up to speed on a variety of exotic and recently introduced microbes in engagingly titled chapters such as "The Birds that Fell from the Sky" (West Nile), "Corona of Death" (SARS), and "Something in the Water" (*Cryptosporidium*). In addition, they describe hantavirus pulmonary syndrome, mad cow disease, and Legionnaires' disease in the context of recent public health emergencies. The authors also explain why both smallpox and anthrax are more than abstract concerns as agents of bioterrorism, on the basis of weaponization history, intrinsic attributes, and realistic scenarios. An account of the 1970 smallpox outbreak, which occurred in Aralsk, Kazakhstan, as a consequence of open air testing of a smallpox weapon by the Soviets is an eye-opener; there should be no doubts about capability and intent after reading this story.

The scenarios are well chosen and informative; they highlight the importance of early recognition that "something has happened" and breaking the disease cycle close to the index case. The unifying theme of the book is the importance of syndrome-based surveillance in achieving this goal. The authors dismiss BIOWATCH (air-monitoring devices to detect and identify microbes in aerosol clouds) as a well-intended but expensive "work in progress," to put it charitably. BIOSENSE is a national surveillance system that they say has not been implemented in any substantive way. I reluctantly find myself in agreement with these assessments and receptive to their suggestions to implement an emerging diseases reporting system based on syndromic reporting.

Healthcare providers recognize syndromes, not microbial diseases. How long did it take to recognize monkeypox in 2003? The hantavirus associated with lethal pulmonary syndrome in New Mexico in 1993 was recognized only when the pattern emerged among previously healthy young adults living in rustic conditions on a Navajo reservation. The authors describe a product they dub Syndromic Reporting Information System (SYRIS) as a "beta test" product that has been deployed on a limited, regional basis and promises to provide a near instantaneous map of syndromic reports and to comply with all Centers for Disease Control and Prevention requirements for electronic reporting systems. Like most good ideas, simplicity is central to the SYRIS concept; it is likely to succeed because participating doctors, nurses, and veterinarians (most of the exotic pathogens are zoonoses) can report syndromic occurrences in 15 seconds or less and will be rewarded with instantaneous feedback and tailored reports and alarms. While this section does read a bit like an infomercial, the concept is sound and worthy of serious consideration by public health officials and policy makers. This book is the best of its genre and is recommended for anyone interested in understanding and managing the risks associated with emerging microbial threats.

